# Understanding the Relations between Soil Biochemical Properties and N_2_O Emissions in a Long-Term Integrated Crop–Livestock System

**DOI:** 10.3390/plants13030365

**Published:** 2024-01-26

**Authors:** Arminda Moreira de Carvalho, Maria Lucrécia Gerosa Ramos, Divina Cléia Resende Dos Santos, Alexsandra Duarte de Oliveira, Ieda de Carvalho Mendes, Stefany Braz Silva, Thais Rodrigues de Sousa, Raíssa de Araujo Dantas, Antonio Marcos Miranda Silva, Robélio Leandro Marchão

**Affiliations:** 1 Embrapa Cerrados, BR-020, Km 18, Planaltina 73310-970, Brazil; alexsandra.duarte@embrapa.br (A.D.d.O.); ieda.mendes@embrapa.br (I.d.C.M.); rahdantas08@gmail.com (R.d.A.D.); robelio.marchao@embrapa.br (R.L.M.); 2 Faculty of Agronomy and Veterinary Medicine, University of Brasilia, Campus Darcy Ribeiro, Brasilia 70910-970, Brazil; cleiadivina@hotmail.com (D.C.R.D.S.); stefany.agrounb@gmail.com (S.B.S.); tharodrigues2506@gmail.com (T.R.d.S.); 3 Department of Soil Science, University of São Paulo-ESALQ, Av. Pádua Dias, 11, Piracicaba 13418-900, Brazil; antoniomarcos@usp.br

**Keywords:** greenhouse gas emissions, microbial biomass, soil enzymes, *Urochloa brizantha*

## Abstract

Edaphoclimatic conditions influence nitrous oxide (N_2_O) emissions from agricultural systems where soil biochemical properties play a key role. This study addressed cumulative N_2_O emissions and their relations with soil biochemical properties in a long-term experiment (26 years) with integrated crop–livestock farming systems fertilized with two P and K rates. The farming systems consisted of continuous crops fertilized with half of the recommended P and K rates (CCF1), continuous crops at the recommended P and K rates (CCF2), an integrated crop–livestock system with half of the recommended P and K rates (ICLF1), and an integrated crop–livestock at the recommended P and K rates (ICLF2). The ICLF2 may have promoted the greatest entry of carbon into the soil and positively influenced the soil’s biochemical properties. Total carbon (TC) was highest in ICLF2 in both growing seasons. The particulate and mineral-associated fractions in 2016 and 2017, respectively, and the microbial biomass fraction in the two growing seasons were also very high. Acid phosphatase and arylsulfatase in ICLF1 and ICLF2 were highest in 2016. The soil properties correlated with cumulative N_2_O emissions were TC, total nitrogen (TN), particulate nitrogen (PN), available nitrogen (AN), mineral-associated organic carbon (MAC), and microbial biomass carbon (MBC). The results indicated that ICLF2 induces an accumulation of more stable organic matter (OM) fractions that are unavailable to the microbiota in the short term and result in lower N_2_O emissions.

## 1. Introduction

On a 100-year timescale, the greenhouse gas nitrous oxide (N_2_O) has a 265 to 298 times greater global warming potential (GWP) than CO_2_. This GHG remains in the atmosphere for 121 years [1,2]. The atmospheric N_2_O concentration has risen significantly since the pre-industrial period, from 270 ppb in 1750 to 330 ppb in recent years [3]. Currently, concentrations continue to increase by 0.73 ppb year^–1^ [4], contributing to climate change. By modeling, Silva et al. [5] predicted such increases in N_2_O emissions for the next five decades, regardless of the crop or soil management system, for the conditions of the Brazilian Cerrado.

Nitrous oxide is the most important greenhouse gas emitted from agricultural soils as a byproduct of microbial nitrification and denitrification [6]. Agriculture and soil management account for 78% of the N_2_O anthropogenic emissions [7]. In Brazil, 87% of the N_2_O emissions are attributed to the agriculture and livestock sector [8]. The main reasons for this high contribution to GHG emissions are organic matter oxidation and microbe-mediated processes, which are sensitive to plant residue management, nitrogen fertilization [9], and climatic conditions [6]. 

Since agriculture contributes significantly to N_2_O emissions, it is imperative to find new solutions to increase soil carbon [10] and crop productivity while reducing the environmental burden of the agricultural system [11]. To this end, in line with the Paris Agreement, the Brazilian government established strategies to deal with climate change in agriculture, one of which is the expansion of integrated crop–livestock system areas [12].

In integrated crop–livestock systems that include crops and pasture, N_2_O emissions are more effectively mitigated than under traditional management. According to Amadori et al. [13], integrated farming systems in subtropical regions based on crop/livestock, livestock/forestry, or crop/livestock/forestry reduced soil N_2_O emissions by 27–40% compared to livestock only. Under similar climate conditions, Pereira et al. [14] showed the potential of integrated agricultural systems to reduce N_2_O losses when compared to monoculture systems. In an experimental area in the tropics, Sato et al. [15], observed that even on clayey Oxisols, integrated systems such as integrated crop–livestock are an alternative to mitigate N_2_O emissions when compared to continuous cropping due to the accumulation of stabilized C fractions in the soil organic matter (SOM), unavailable to the microorganisms. Based on these recent studies, the potential of integrated farming systems to mitigate soil N_2_O emissions is greater than that of livestock/monoculture systems.

Soil use and management influence microbial nitrification and denitrification, which are biogenic sources of N_2_O emissions [16,17,18]. In addition, soil microbiological properties play an important role in C and N dynamics, but the influence on N_2_O emissions requires further investigation, mainly in diversified systems such as crop/livestock farming. The microbiological properties that represent C and N dynamics are microbial biomass and soil enzymatic activity.

Microbial biomass (MB) is a labile fraction of soil organic matter sensitive to changes in soil management [9,19]. Phosphorus and K fertilization can also change the MB. Huang et al. [20] observed greater microbial abundance after two years of fertilization with high P rates (30 g P m^−2^ yr^−1^) compared to lower rates. The authors explained this increase in MB by an increase in soil organic carbon. A few studies have addressed the significant correlation between MB and N_2_O fluxes [9,15,21].

Most biochemical transformations are mediated by enzymes and influenced by soil management. In other words, the soil enzymatic activity is a sensitive indicator of land use change [22,23]. The determination of soil enzymatic activity allows an evaluation of the impact of management practices on soil microbiota and, consequently, on N mineralization and N_2_O emissions. 

Few studies have assessed the changes in soil C fractions promoted by soil management and their relationship with N_2_O fluxes [15,24]. The soil particulate fractions of C and N are associated with the formation and stabilization of soil aggregates [24,25,26]. Therefore, these fractions (>53 μm) are sensitive to land use change. They play an important role in nutrient cycling and, consequently, N_2_O emission [9]. Not many studies have related soil carbon fractions with N_2_O emissions, but in a recent study, Sato et al. [15] showed that a high humic acid concentration reduced N_2_O emissions. The authors observed that in crop–livestock systems under no-tillage, with *Urochloa* grasses, the highest organic carbon contents in soil microaggregates were associated with lower cumulative N_2_O emissions. 

Studies that correlate N_2_O fluxes and soil properties are scarce [9,21], and no information about soil enzymatic activity and cumulative N_2_O fluxes could be found, in particular for crop–livestock integration systems. Our main hypothesis is that biochemical properties, such as soil enzymes and SOM fractions, are indicators of the N_2_O mitigation potential in diversified systems with forages and crops, such as integrated crop–livestock farming systems. These integrated agricultural systems represent a promising option for the Brazilian public policy of low-C agriculture to mitigate GHGs and global climate change. In this context, it is essential to deepen the understanding of the relationships between soil biochemical properties and N_2_O emissions in long-term integrated crop–livestock systems.

The objective of this study was to evaluate cumulative N_2_O emissions and their relationships with soil biochemical properties in a two-year phase with soybean in a long-term experiment (26 years) with integrated crop–livestock farming systems fertilized with two P and K rates.

## 2. Results

### 2.1. Soil C and N

The data of soil total carbon (TC), particulate carbon fraction (PC), and mineral-associated organic carbon (MAC) in the treatments are listed in Table 1. Total carbon levels were higher in ICLF2 than in the other treatments in the first and second years of evaluation. In the first year, the PC content in ICLF2 was 56% higher than in CCF1. In the second year of evaluation, there were no differences between the systems, and the mean PC was 2.85 g kg^−1^. Mineral-associated organic carbon (MAC) was similar between management systems in the first year, and in the second, the value was higher in ICLF2 than in ICLF1 and CCF2.

Data on soil total nitrogen (TN), particulate nitrogen (PN), mineral-associated total nitrogen (MAN), and available nitrogen (AN) in the treatments are listed in Table 2. With regard to TN, the values in the first year were higher in CCF2 than in CCF1 and ICLF2. In the second year, TN was similar in both management systems (mean of 2.39 g kg^−1^) (Table 2). For PN, there was no significant difference between production systems in the two years (*p* > 0.05). In the first year of evaluation, MAN was higher in CCF2, although not significantly different from either ICL treatment, while in the second year, the management systems did not differ. In the first year, AN did not differ between management systems but was higher in CCF2 than CCF1, ICLF1, and ICLF2 in the second year.

The chemical carbon fractions of fulvic acid (C-FA), humic acid (C-HA), and humin (C-HUM) were similar between the production systems in both years of evaluation, except in the second year in C-HA when there were differences between CC and ICL treatments (Table 3). The mean C-FA content was 7 and 7.5 g kg^−1^ in the first and second years, respectively, and the mean C-HA content was 1.55 and 2.24 g kg^−1^ in the first and second years, respectively. The mean C-HUM content in the first and second years was 8.93 and 10.36 g kg^−1^, respectively.

### 2.2. Microbial Biomass C and N

For microbial biomass carbon (MBC), the management systems differed in the first and second years of evaluation (Table 4). In the first year, the systems with the highest MBC were ICLF2 and CCF2. In the second year, MBC was highest in the treatment of ICLF2. In both years of evaluation, MBC was lowest under CCF1. Microbial quotient carbon (MQC) was higher under CCF2 than CCF1 in the first year, and in the second, CCF1 had the lowest values. 

For microbial biomass nitrogen (MBN), the management systems differed in both study years (*p* < 0.05). In the first year, MBN was higher under CCF1 than CCF2 and ICLF1. In the second year, the MBN was higher under ICLF2 than CCF1. In the first year, MQN was higher under CCF1 than CCF2 and ICLF1. In the second year, there was no significant difference between management systems. 

### 2.3. Cumulative N_2_O Emissions and Soil Enzymes

Cumulative emissions in the first growing season (2015/2016) were higher under CCF1 and CCF2 than ICLF2 and ICLF1 (Table 5). In the second season (2016/2017), there was no significant difference between management (mean cumulative N_2_O fluxes of 0.17 kg ha^−1^).

**Figure 1:**
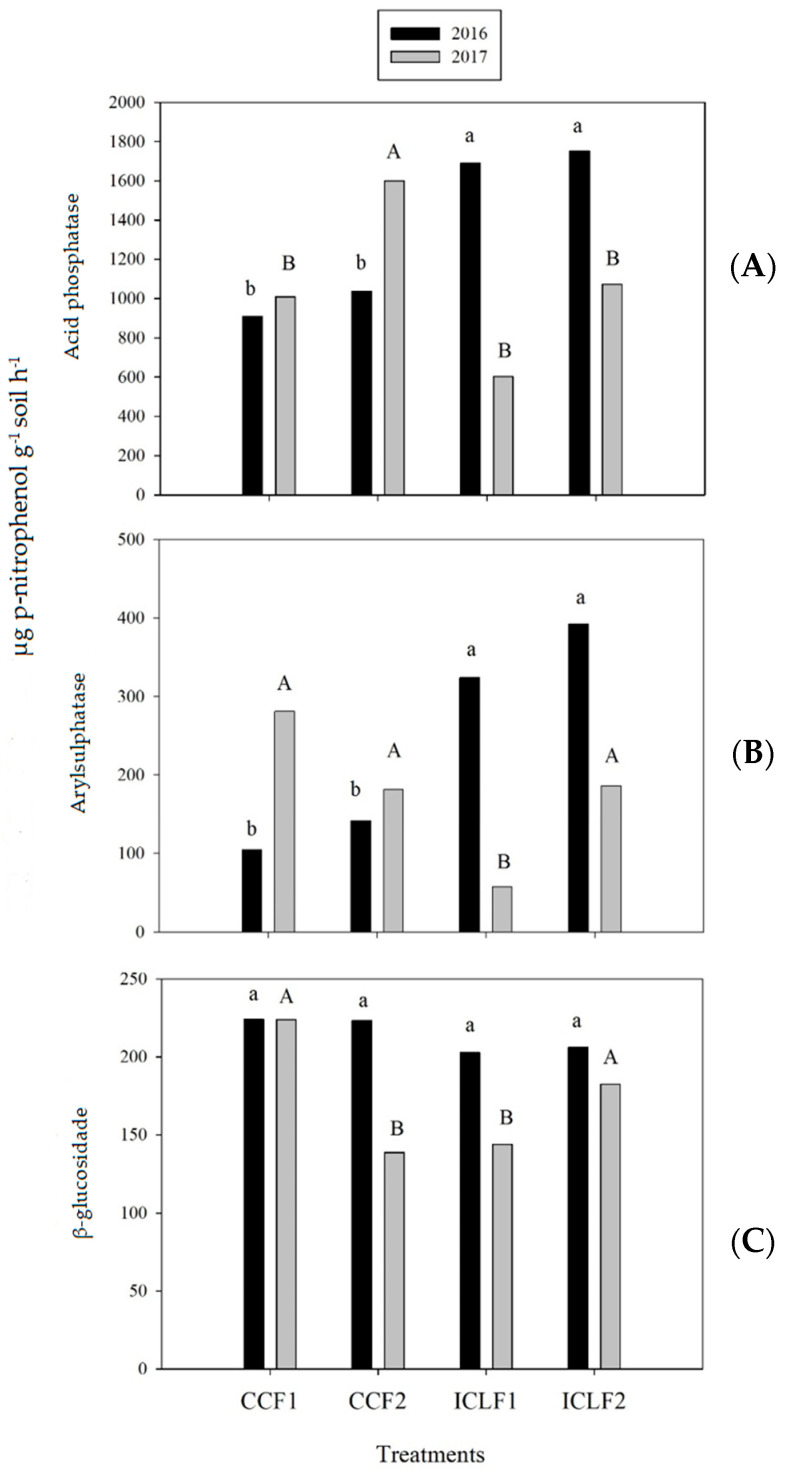
Enzyme activity. (**A**) Acid phosphatase, (**B**) arylsulfatase, and (**C**) ß-glucosidase at soybean flowering in 2016 (black columns) and 2017 (gray columns) under different management systems. CCF1: continuous crops fertilized with half the recommended P and K rates; CCF2: continuous crops fertilized with the full recommended P and K rates; ICLF1: integrated crop–livestock with half the recommended P and K rates; ICLF2: integrated crop–livestock at full recommended P and K rates. Means followed by the same letter in a column did not differ by the Tukey test at 5% probability.

Acid phosphatase and arylsulfatase activities were highest under ICLF1 and ICLF2 in the first year of evaluation, while in the second, acid phosphatase was higher in CCF2, and arylsulfatase was similar in all treatments except ICLF1 (Figure 1A,B). 

For β-glucosidase activity, there was no difference between the management systems in the first year of evaluation (Figure 1C). In the second year, the mean values of β-glucosidase activity were highest in the treatments CCF1 and ICLF2.

**Figure 2:**
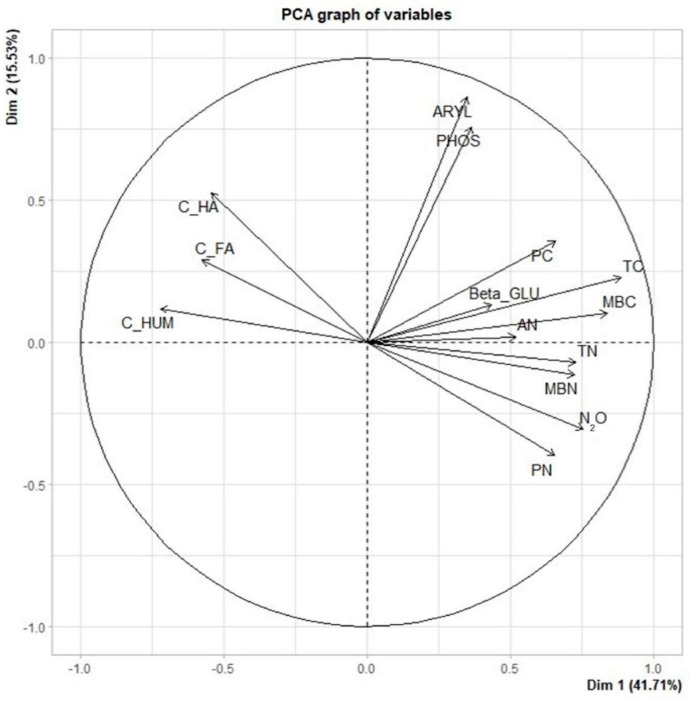
PCA biplot of PC1 and PC2 scores; arrows indicate the weights of the biological and chemical variables.

Principal component analysis indicated that 57.2% of the original data could be explained by the two principal components in both years of evaluation (Figure 2).

According to the PCA, TC was positively correlated with all variables except AN and soil organic matter fractions. Total N was positively correlated with PN, MBC, MBN, and cumulative N_2_O emissions. The soil enzymes PHOS and ARYL were positively correlated. Aside from TC and TN, the variables that correlated positively with cumulative N_2_O emissions were PN, AN, MBC, and MBN.

The biological activity, represented by enzyme activity and the labile fractions of C and N represented by MBC, MBN, AN, PC, and PN, and cumulative N_2_O emissions (N_2_O), are biological and chemical variables that indicate soil C and N transformation (mineralization and nitrification). Conversely, the soil organic matter fractions (C-HA, C-FA, and C-HUM) are related to soil organic matter stabilization. 

**Figure 3:**
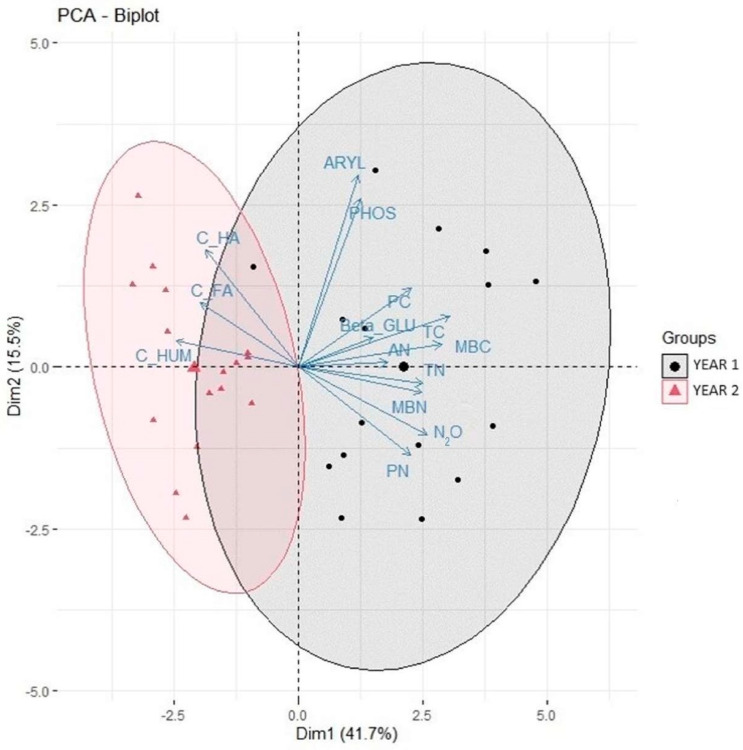
PCA biplot of PC1 and PC2 scores; arrows indicate the weights of the biological and chemical variables and points indicate the years of evaluation (YEAR 1 = 2016, YEAR 2 = 2017).

Principal component analysis for the effect of years of evaluation highlighted the effect of time on soil organic matter stabilization (Figure 3).

## 3. Discussion

### 3.1. Effect of Management Systems on Soil C and N

At soil sampling in the growing seasons 2015/2016 and 2016/2017, all production systems were in the cropping phase with soybean, whereas in the previous 26 years, other crops and intercrops had been grown (Table 6).

Leaving plant residues on the soil surface in integrated production systems may have contributed to soil C accumulation, especially in ICLF2, which was treated with corrective P and K rates from 1995 to 2013. This fertilization may have contributed to increased crop and forage root growth during the experimental period and, therefore, to the rise in soil C levels, as observed by Carvalho et al. [27] in the same experimental area.

In agreement, in a long-term crop–livestock integration system, Soares et al. [28] observed the highest total C in the 0–10 cm layer (40 g kg soil^−1^). The authors attributed this to the constant addition of plant residues on the soil surface (around 10 t ha^−1^) in addition to animal grazing in the area.

The management system ICLF2 resulted in a higher PC fraction (>53 μm) than CCF1 in the first year of evaluation. This fraction is associated with the formation of soil aggregates [24,25,26] and is a labile compartment of soil organic matter, highly sensitive to soil management [29]. The MAC fraction was much higher than particulate carbon (PC) in all production systems, indicating the strong interaction of organic C with Fe and Al oxides [30].

In the second year of evaluation, the PC fraction was similar in both production systems and MAC was higher under ICLF2 but not significantly different from CCF1. These data suggested that the physical fractions of soil organic matter are dynamic and can be altered according to the management system and C input into the soil within a short period (one season).

Total N was higher in CCF2 (5.51 g kg^−1^) in the first year of evaluation, probably due to the combination of the effects of crop residues from previous years (maize and sorghum), soybean root mineralization, and the higher N fertilization rates in this treatment.

**Figure 4:**
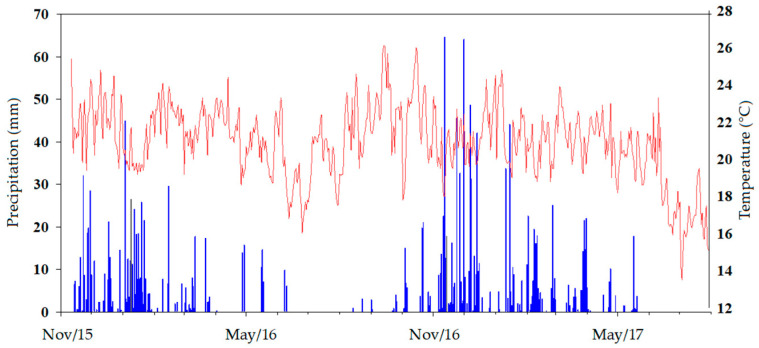
Average precipitation and temperature in the experimental area (2015–2017). Blue color is precipitation and red is temperature.

In the second year of evaluation, TN did not differ between the production systems, probably due to changes in the amount of plant residue input and roots in the subsurface. The lower biomass accumulation can be attributed to a period of drought at the beginning of 2016 (184 mm in 186 days) (Figure 4) [31].

The variation in soil TN seems to be directly affected by the turnover of the MAN fraction. Recent studies indicated that this fraction is strongly affected by changes in soil management and can be more easily accessed by microorganisms [32,33,34]. Moreover, the MAN turnover is driven by root exudates that contribute to N release [35].

The particulate nitrogen fraction (PN) was similar in both years of evaluation for all production systems, i.e., it was less sensitive to management and environmental factors than the particulate carbon fraction (PC). 

Mineral-associated total nitrogen (MAN) was higher in CCF2 but did not differ from the ICL systems in the first year. According to Skol and Bradford [36], MAN formation depends on differences in aboveground or belowground residue inputs. In this experiment, the continuous crop system contributes to a high input of readily decomposable residues, but on the other hand, the integrated crop–livestock system diversification contributed to root exudate inputs, especially by *Urochloa* species [37]. Mineral-associated C and N are considered critical factors and major mediators of C and N bioavailability in the rhizosphere. Some authors observed that C and N association occurs mainly in integrated cultivation and systems with high nutrient input [38,39].

Available nitrogen (AN) is a fraction composed of nitrate, in addition to readily decomposable organic forms such as amino sugars and amino acids [40], and the variation between the years of evaluation can be attributed to this rapid turnover. In the first year of evaluation, the treatments did not differ despite the lower N application in the integrated system. The lack of difference can be attributed to diversification in integrated systems, especially with the introduction of *Urochloa* grasses. *Urochloa* species can reduce nitrogen losses by nitrification inhibition and recover nutrients leached through the soil profile [37]. In the second year of evaluation, the AN content decreased when compared to 2016 in CCF1, ICLF1, and ICLF2 but did not decline in CCF2. This pattern can be attributed to the low input of plant residues due to drought stress in the season and the differences between treatments in N fertilization rates.

### 3.2. Effect of Management Systems on Biomass C and N

Although MBC represents 1 to 3% of the total carbon (TC), it is the most active and important soil fraction, which is mineralized and promotes nutrient cycling [41]. The production systems influenced microbial biomass C and N. The MBC in ICLF2 was higher than in the other treatments in both years of evaluation, showing that the alternating P and K fertilization between 1995 and 2013 along with the integrated system may have modified MBC in 2016 and 2017. These data may indicate that, even three years later with the same PK fertilization in all production systems, the previous period of higher PK rates still influenced MBC. It is worth mentioning that the germination failure of the cover crops after the rainy season drastically affected MBC at soybean flowering in 2017. Under ICLF2, the MBC was 939.89 mg kg^−1^ in 2016 and 360 mg kg^−1^ in 2017.

Microbial biomass may be responsible for greater P mineralization, resulting in an increase in this nutrient in the soil [42,43], which may be one of the reasons why MBC was approximately twice as high under CCF2 and ICLF2 as it was under CCF1 and ICLF1. Other authors also found higher MBC in integrated systems [22,44]. For a crop–livestock integration system (*Urochloa humidicola* intercropped with corn), Coser et al. [19] also reported high MBC values (around 200 mg C kg^−1^) three years after the implementation of the management system. These values are much lower than those determined in this study, installed for 26 years, with MBC between 488.33 and 939.89 mg C kg^−1^ in 2016. This indicates that the combination of different plant species, the presence of animals in the areas, and the age of the production system possibly promoted an increase in MBC, as well as C accumulation in the soil over the years, mainly in the ICLF2 system.

On the other hand, in the second year of evaluation, the MBC values decreased, probably due to the dry spell after the rainy season, and MBC decreases under drought stress have been reported elsewhere [45]. Nevertheless, MBC was highest under ICLF2 (360.4 mg C kg^−1^ soil). According to Lopes et al. [22], adequate MBC values for healthy soil in the Cerrado biome, for the same soil class as in this experiment, must be higher than 450 mg kg^−1^. In this study, in the first year of evaluation, the values in all production systems exceeded those suggested by the above authors, whereas, in the second year, none of the production systems reached adequate MBC values.

The microbial quotient carbon (MQC) ranged from 2.60 to 1.47%, respectively, in CCF2 and CCF1 in 2016. The MQC is an indicator of soil quality [28] that is influenced by several factors, e.g., the stabilization degree of organic C and substrate availability for microorganisms [46]. High microbial quotients, as in the case of CCF1, ICLF1, and ICLF2, indicate an intensified incorporation of total soil organic carbon in the microbial biomass [47], which enhances the quality of soil organic matter. In addition, the reduction in MQC in 2017 indicated a decrease in microbial immobilization of soil organic carbon, which may be related to environmental factors, such as rainfall distribution in the second year of evaluation. Similar results were reported by Lepcha and Devi [48] in a two-year evaluation of MQC, where the quotient decreased due to lower rainfall distribution in the second year.

Similarly, as in the case of MBC, MBN was also drastically reduced by the water stress in 2017. In the ICLF2 treatment, MBN had high values in both years of evaluation, with similar values to CCF1 and ICLF1 in the first year and CCF2 and ICLF1 in the second. Thus, different plant species with different root architecture exudate different amounts of organic compounds that are sources of C and energy for soil microorganisms [49,50]. In addition, unfavorable environmental conditions such as those that affected this study seem to have hampered the soil microbiological indicators.

### 3.3. Effect of Management Systems on Cumulative N_2_O Emissions and Soil Enzymes

Cumulative N_2_O emissions during the soybean cycle in the 2015/2016 and 2016/2017 seasons are shown in Table 5. In the 2015/2016 season, cumulative N_2_O emissions were highest from CCF1 and CCF2. These results may be associated with the amount of N (provided by soybean roots and N-rich nodules) in addition to the fertilization history, with higher N fertilization than in the ICL treatments [51]. Forage crop rotation has been described as a N_2_O-mitigating system, with lower N_2_O emissions than from other production systems, as observed by Carvalho et al. [27] under similar experimental conditions.

Soil enzymes play an important role in mineralization and nutrient cycling. Therefore, increased enzymatic activity is an indicator of organic matter accumulation and efficient C, N, P, and S cycling [52,53]. Under ICLF1 and ICLF2, the soil activities of acid phosphatase and arylsulfatase were higher in 2016, indicating that grass cultivated previously in the area influenced both years of soybean cultivation and had greater enzyme activity than CCF1 and CCF2. These results reinforced the importance of integrated production systems, including diverse species and the presence of animals in the area.

β-glucosidase activity has often been reported to be very sensitive to management practices [54,55]. The results in this study were different, mainly in the first year of evaluation, when no significant difference was detected between the soil management systems. In general, as well as for the other soil microbiological properties, the enzyme activity decreased in 2017. This decrease can be attributed to the lower input of plant residues due to the drought stress in that growing season. The variation in plant residue availability promoted changes in the soil microbial community and enzyme activities [56].

In the context of global warming, it is important to understand how extreme climate events affect the functionality of ecosystem services such as decomposition and nutrient cycling [57] and to propose management strategies to minimize the negative impacts of greenhouse gas emissions and consequently reduce the impact on climate change.

### 3.4. Relationship between Soil Properties, Cumulative N_2_O Emissions, and Soil Enzymes

As shown in Table 5, cumulative N_2_O emissions were influenced by the management system in the first year of evaluation. Emissions were higher from the CC than the ICL systems. However, in the second year of evaluation, emissions did not differ between the management systems.

Crop residue input can affect N_2_O emissions, especially in the CC system, where the low C/N ratio residues can provide easily decomposable C for nitrifying/denitrifying bacteria. In the ICL system, the introduction of pasture crops, particularly grasses (*Urochloa* species) with a higher C/N ratio, reduced N_2_O emissions compared to the CC system [11].

In the same experimental area, Sato et al. [15] observed that cumulative N_2_O emissions from ICL systems were lower than from the CC system. In the ICL systems, the authors found higher C accumulation in most stable soil organic matter fractions that were not readily available to microorganisms. In addition, Carvalho et al. [27] evaluated seasonal N_2_O emissions in 2016/2017 and found lower daily N_2_O fluxes from ICLF1 and ICLF2.

The variables that mainly affect cumulative N_2_O emissions were highlighted by PCA in a multivariate model. The significant correlation between cumulative N_2_O emissions and PN (r = 0.65) showed that the N particulate fraction is more vulnerable to disturbance and that nutrient cycling was accelerated and thus favored N transformations [58]. As expected, cumulative N_2_O emissions were positively correlated with AN [59]. 

The cumulative N_2_O emissions were also positively correlated with MBC and MBN. Soil microbial biomass is a soil organic matter fraction with poor stability, fast turnover rate, and easy mineralization and decomposition, with a direct influence on N_2_O production and emission [60]. 

No correlation between the soil enzyme activity under study with cumulative N_2_O emissions was observed in this experiment.

## 4. Materials and Methods

### 4.1. Site Description and Experimental Design

The experiment was carried out in a rainfed experimental area of Embrapa Cerrados, Planaltina, Federal District (15°39′ S, 47°44′ W; 1200 m asl). The climate was classified as Aw (tropical savannah), with a dry (winter) and rainy period (summer, October to April), according to Köppen–Geiger’s classification [61]. Precipitation and temperature data from 2015 to 2017 are shown in Figure 4.

The soil was classified as a clayey Oxisol based on Embrapa [62]. 

The long-term experiment was initiated in 1991 on 40 × 50 m (2000 m^2^) plots. At that time, 5.8 Mg ha^−1^ of limestone was applied to achieve 50% base saturation. As a side dressing, 20 kg P_2_O_5_ ha^−1^, 50 kg K_2_O ha^−1,^ and 60 kg N ha^−1^ were applied. In the first four years, soybean, maize, rice, and sorghum were cultivated in the area in a conventional system (disk and moldboard plow). 

Four years after the implementation of the experiment, *Andropogon gayanus* was sown in the area. In the continuous pasture treatment, 20 kg ha^−1^ P_2_O_5_, 50 kg ha^−1^ K_2_O, and 60 kg ha^−1^ N were applied as side dressing. In the integrated crop–livestock treatment, no fertilizers were applied in the pasture phase, since enough residual fertilizer from the previous crop was still available for the growth of *Andropogon gayanus*. In the continuous crop treatment and the crop phase of the integrated crop–livestock system, gradual liming was applied according to the technical recommendations for each crop. 

Between 1995 and 2013, the plots were fertilized with the full or half the recommended rate of potassium and phosphorus fertilizers. The experiment was laid out in a randomized block design (two blocks) with a 2 × 2 factorial arrangement (farming systems × P and K rates). The treatments (plots) consisted of continuous crops fertilized with half of the recommended P and K rates (CCF1), continuous crops with the full recommended P and K rates (CCF2), an integrated crop–livestock system with half the recommended P and K rates (ICLF1), and an integrated crop–livestock system at full recommended P and K rates (ICLF2).

The sequence of the crop systems throughout the 26 years is listed in Table 6.

Soybean cv. BRS 8180 RR was sown in November 2015 of the 2015/2016 growing season and harvested in March 2016, and cv. NS 7200 RR in November 2016 of the 2016/2017 growing season and harvested in February 2017. The soil chemical properties of each treatment were analyzed in January 2016 (Table 7).

Soybean seeds were inoculated with *Bradyrhizobium japonicum* and fertilization consisted of 115 kg P_2_O_5_ ha^−1^ and 100 kg K_2_O ha^−1^ in both growing seasons (2015/2016 and 2016/2017). The forage was fertilized with 10 kg N ha^−1^, 50 kg P_2_O_5_ ha^−1^, and 30 kg K_2_O ha^−1^.

### 4.2. Soil Sampling and Analysis

The soil was sampled at soybean flowering in January 2016 and January 2017 (0–10 cm layer) because our objective was to evaluate biochemical properties under optimal conditions of biological activity (rainy season). Five subsamples were taken per plot to blend one composite sample. Part of the samples was stored in an ice box and placed in a refrigerator (±4 °C) for posterior microbiological analysis, and the other part was dried at room temperature for chemical analysis.

The biological and chemical analyses measured soil total C and total N (TC and TN); soil particulate C and N fractions (PC and PN); mineral-associated C and N (MAC and MAN); soil microbial biomass C (MBC) and N (MBN); C and N microbial quotient (CMQ and NMQ); soil available nitrogen (AN); humic fractions of organic C; fulvic-acid (C-FA); humic acid (C-HA); humin (C-HUM); and the soil enzymes β-glucosidase (β-GLU), arylsulfatase (ARYL), and acid phosphatase (PHOS).

#### 4.2.1. Soil Total C and N

The soil samples dried at room temperature were sieved (<2 mm/10-mesh) and ground and sieved through a 0.149 mm/100-mesh sieve. Soil TC and TN were determined in a CHNS/O analyzer (Perkin Elmer 2400 Series II).

#### 4.2.2. Soil Particulate C and N Fractions

Carbon and N were determined by soil physical fractionation, as proposed by Cambardella and Elliott [63], with modifications suggested by Bayer et al. [64] and Bongiovanni and Lobartini [65]. Soil samples dried at room temperature were sieved (<2 mm/10-mesh) and 20 g of soil was placed in glass tubes with 70 mL of 0.5% sodium hexametaphosphate and shaken (150 rpm) for 15 h in a horizontal shaker. The suspension was sieved through a 270-mesh sieve (<53 µm) and washed thoroughly by spraying with water. The soil retained in the sieve was oven-dried at 45 °C and ground for analysis of total C and N in a CHNS/O analyzer (Perkin Elmer 2400 Series II). The fractions MAC and MAN were calculated by the difference between TC and PC and between TN and PN, respectively.

#### 4.2.3. Soil Microbial Biomass C and N

Microbial biomass carbon and MBN were determined by the fumigation/extraction method [66,67,68]. Soil samples were sieved through an 8 mm sieve to remove roots and plant residues. Prior to fumigation, the sample moisture content was corrected to 80% of the soil retention capacity. The samples were divided into six subsamples of 20 g and incubated for seven days. Half of the samples were fumigated with ethanol-free chloroform for 24 h.

Carbon and nitrogen extraction was performed by adding 5 mol L^−1^ K_2_SO_4_ to fumigated and non-fumigated samples, which were shaken in a continuous shaker at 150 rpm for 40 min and then filtered. Ten mL of extracted solution and 1 mL of substrate were added to glass tubes with 1 mL of distilled water. Microbial biomass carbon and MBN were determined in a CHNS/O analyzer (Perkin Elmer 2400 Series II).

The MBC and MBN contents were determined as the difference between carbon and nitrogen contents in fumigated and non-fumigated samples using a correction factor of Kc = 0.35 and Kn = 0.54 for MBC and MBN, respectively, according to Joergensen [69]. The MQC and MQN were calculated as the ratio of MBC or MBN by TC or TN, respectively, and expressed as percentages.

#### 4.2.4. Chemical Fractionation of Soil Organic Matter

Soil organic matter was chemically fractionated using 0.1 mol L^−1^ NaOH as the extractor (10:1). The fractionation products were the three fractions of soil organic matter: humic acid (C-HA), fulvic acid (C-FA), and humin (C-HUM), determined according to their solubility in basic and acid solution. The C-HUM precipitates in a basic solution (insoluble in basic pH). The fractions C-HUM and C-FA were obtained by extract acidification with 6 mol L^−1^ HCl at pH 1.0. The precipitate (C-HUM) and supernatant (C-FA) were separated by centrifugation (4500 rpm) for 30 min [70]. Total organic C was determined by humic oxidation of organic matter with potassium dichromate in sulfuric acid [71].

#### 4.2.5. Soil Available N

Soil available N was determined, as suggested by Gianello and Bremner [72], by measuring the ammonia-N produced from soil organic N after steam sterilization of the soil with Na_3_PO_4_-borate buffer (pH 11.2). Two grams of soil were transferred to glass tubes with 25 mL of pH 11.2 Na_3_PO_4_-borate buffer solution (200 g Na_3_PO_4_·12H_2_O + 50 g borax in 2000 mL of distilled water), 0.23 g MgO, 0.1 g Devarda’s alloy, and 10 drops of dimethicone (to reduce foam formation), and the tubes were placed in a micro-distiller.

The distillate was filled into a 50 mL volumetric flask containing 10 mL of 0.05 mol L^−1^ HCl. For the calculations, we used a calibration curve obtained by distillation of standard N solutions containing 0, 10, 25, 50, 75, and 95 μg N mL^−1^. The extracted N was determined with a spectrophotometer (Tecnal SP 2000 UV) at 440 nm.

#### 4.2.6. Soil Enzymes 

The activities of the soil enzymes acid phosphatase (PHOS), β-glucosidase (β-GLU), and arylsulfatase (ARYL) were determined according to Tabatabai [73]. The methodology is based on the colorimetric determination of p-nitrophenol released after soil incubation at 37 °C for 1 h in a specific substrate. For each sample, two analytical repetitions plus a control (no enzyme addition) were used.

The soil samples separated to determine the β-glucosidase and arylsulfatase activities, previously stored at 4 °C, were dried and sieved, as proposed by Lopes et al. [74]. To determine the amount of p-nitrophenol released from the samples, a standard curve was prepared with known p-nitrophenol concentrations (0, 10, 20, 30, 40, and 50 µg p-nitrophenol mL^−1^). The enzyme activities were expressed in µg p-nitrophenol h^−1^ g^−1^ soil.

#### 4.2.7. Nitrous Oxide Sampling and Analysis

The N_2_O fluxes were evaluated during soybean growth in the growing seasons of 2015/2016 and 2016/2017. In the total evaluation period, 78 samplings were carried out in 603 days [27]. After sowing and N fertilization, N_2_O fluxes were measured for up to five consecutive days. After rainfall, tilling, or harvesting, N_2_O fluxes were measured for 2–3 consecutive days. During the dry season, N_2_O fluxes were evaluated every 15 days. Several calibration measurements were carried out in the same experimental area [15,27].

The N_2_O fluxes were measured in closed static chambers using the methodology described by Sato et al. [75]. On each plot, two static chambers were maintained from November 2015 to July 2017, one installed in the planting row and the other in between the rows. Each chamber consisted of a rectangular hollow metal frame (38 × 58 × 6 cm) inserted to a depth of 5 cm into the soil. The top of each chamber was covered with a polyethylene tray coupled to the base during gas sampling. To avoid airtightness during N_2_O sampling, soft rubber was added and both ends were fixed to the metal base. A three-way Luer valve was attached to the top part of the tray to fasten the syringes for gas sampling. The N_2_O samples were collected in 60 mL polypropylene syringes and transferred to 20 mL pre-evacuated glass vials.

The N_2_O concentration was determined by gas chromatography (Thermo Scientific Model Trace 1310, Milan, Italy), with “Porapak Q” columns comprising a backflush system connected to an electron capture detector. The N_2_O fluxes (FN_2_O) were measured based on the linear variation in gas concentration in relation to incubation time in the chambers, calculated with the following equation:

FN_2_O = [(δC/δt) × (V/A) × (M/Vm)],

where δC/δt is the change in N_2_O concentration in the chamber during the incubation period; V and A are the chamber volume and the covered soil area, respectively; M is the molecular weight of N_2_O; and Vm is the molecular volume at sampling temperature.

Cumulative emissions were estimated by plotting the mean N_2_O fluxes and time scale on a graph and calculating the resulting area under the integration curve using Sigmaplot^®^ Version 10 (Systat Software Inc., Chicago, IL, USA, 2007). The cumulative N_2_O fluxes from each plot were estimated by the integrated trapezoidal area of the daily N_2_O flux over time, considering that fluxes change linearly between measurements [76].

### 4.3. Statistical Analysis

The data were tested for normality of residuals and homogeneity of variances and then subjected to one-way ANOVA using the R program.

The effects of farming systems on cumulative N_2_O fluxes were compared using Tukey’s test (*p* < 0.05). In the model, a nested design was assumed for each land use. The data on chemical and microbiological soil properties were statistically analyzed by the Tukey test (*p* < 0.05). The interaction between years of evaluation was not analyzed due to the drought stress that affected the 2016/2017 growing season.

Prior to principal component analysis (PCA), measures of sampling adequacy were calculated by the Kaiser–Meyer–Olkin (KMO) and Bartlett’s sphericity tests (*p* < 0.05). When the KMO was higher than 0.5–1.0, the PCA application was considered acceptable [77]. Bartlett’s sphericity test checks the independence of the tested variables. Principal component analysis was performed to group the dataset into new variables that resume the information in principal components (PC). The analysis also helps to avoid multicollinearity between the original variables. Principal component analysis (PCA) was performed using the R program and was applied to a data matrix with 16 rows corresponding to the cultivation systems (CCF1, CCF2, ICLF1, and ICLF2 in four repetitions) and 17 columns representing soil properties (TC, TN, PC, PN, MAC, MAN, MBC, MBN, MQC, MQN, AN, C-FA, C-HA, C-HUM, β-GLU, ARYL, and PHOS) to identify which properties most affected cumulative N_2_O fluxes, represented by cumulative emissions.

## 5. Conclusions

The TC values were highest in ICLF2 in both growing seasons, and the particulate and mineral-associated fractions were highest in 2016 and 2017, respectively. Also, the microbial biomass fraction was highest in ICLF2 in both years. In terms of enzyme activity, ICLF1 and ICLF2 stood out with higher acid phosphatase and aryl sulfatase contents.

The soil properties correlated with cumulative N_2_O emission were TC, TN, PN, AN, MBC, and MBN. The integrated systems induced C storage in more stable fractions and reduced available N, decreasing N_2_O emissions and promoting lower cumulative N_2_O emissions, which could mitigate N_2_O.

The production systems ICLF1 and ICLF2 were more closely correlated with soil enzymatic activity (β-glucosidase, arylsulfatase, and acid phosphatase), particulate fraction of soil organic matter, and mineral-associated organic carbon in both growing seasons, whereas enzyme activity was not correlated with N_2_O emissions. In addition, ICLF2 promoted an accumulation of C in the more stable soil organic matter (SOM) fractions, which are unavailable to the microorganisms and resulted in lower N_2_O emissions.

## Figures and Tables

**Table 1:** Soil total carbon (TC), particulate carbon fraction (PC), and mineral-associated organic carbon (MAC) in soil under four management systems.

2016			
Management System	TC	PC	MAC
	g kg^−1^		
CCF1	33.50 b	2.80 b	30.70 a
CCF2	33.27 b	3.86 ab	29.41 a
ICLF1	33.15 b	4.07 ab	29.07 a
ICLF2	39.15 a	6.45 a	32.70 a
2017			
CCF1	27.92 ab	2.65 a	25.27 ab
CCF2	25.82 b	2.52 a	23.30 b
ICLF1	27.35 b	2.82 a	24.52 b
ICLF2	30.92 a	3.40 a	27.85 a

CCF1: continuous crops fertilized with half the recommended P and K rates; CCF2: continuous crops fertilized with the full recommended P and K rates; ICLF1: integrated crop–livestock with half the recommended P and K rates; ICLF2: integrated crop–livestock at full recommended P and K rates. Means followed by the same letter in the columns did not differ by the Tukey test at 5% probability.

**Table 2:** Total nitrogen (TN), particulate nitrogen (PN), mineral-associated total nitrogen (MAN), and available nitrogen (AN) in soil under four management systems.

2016				
Management System	TN	PN	MAN	AN
	g kg^−1^			mg kg^−1^
CCF1	3.45 b	0.35 a	2.82 b	58.81 a
CCF2	5.15 a	0.37 a	4.45 a	53.73 a
ICLF1	3.95 ab	0.35 a	3.32 ab	37.07 a
ICLF2	3.85 b	0.43 a	3.32 ab	65.72 a
2017				
CCF1	2.35 a	0.35 a	2.00 a	29.39 b
CCF2	2.50 a	0.38 a	2.12 a	66.80 a
ICLF1	2.22 a	0.35 a	1.87 a	30.52 b
ICLF2	2.50 a	0.42 a	2.08 a	33.06 b

CCF1: continuous crops fertilized with half the recommended P and K rates; CCF2: continuous crops fertilized with the full recommended P and K rates; ICLF1: integrated crop–livestock with half the recommended P and K rates; ICLF2: integrated crop–livestock at full recommended P and K rates. Means followed by the same letter in the columns did not differ by the Tukey test at 5% probability.

**Table 3:** Carbon fractions of fulvic acid (C-FA), humic acid (C-HA), and humin (C-HUM) in soil under four management systems.

2016			
Management System	C-FA	C-HA	C-HUM
	g kg^−1^		
CCF1	6.99 a	1.57 a	8.83 a
CCF2	7.10 a	1.55 a	9.41 a
ICLF1	7.11 a	2.24 a	9.65 a
ICLF2	6.69 a	1.48 a	7.82 a
2017			
CCF1	7.62 a	2.76 a	11.17 a
CCF2	7.95 a	2.42 a	10.62 a
ICLF1	6.92 a	1.62 b	10.38 a
ICLF2	7.36 a	1.64 b	9.85 a

CCF1: continuous crops fertilized with half the recommended P and K rates; CCF2: continuous crops fertilized with the full recommended P and K rates; ICLF1: integrated crop–livestock with half the recommended P and K rates; ICLF2: integrated crop–livestock at full recommended P and K rates. Means followed by the same letter in the columns did not differ by the Tukey test at 5% probability.

**Table 4:** Microbial biomass carbon (MBC), microbial biomass nitrogen (MBN), microbial quotient carbon (MQC), and microbial quotient nitrogen (MQN) in soil under four management systems.

2016				
Management System	MBC	MBN	MQC	MQN
		mg kg^−1^		%
CCF1	488.33 c	118.9 a	1.47 b	3.42 a
CCF2	855.86 ab	47.35 c	2.60 a	0.91 c
ICLF1	595.72 bc	61.20 bc	1.80 ab	1.57 bc
ICLF2	939.89 a	89.86 ab	2.40 ab	2.54 ab
2017				
CCF1	185.06 c	26.75 b	0.66 b	1.14 a
CCF2	247.42 b	30.35 ab	0.96 a	1.22 a
ICLF1	256.54 b	28.58 ab	0.94 a	1.28 a
ICLF2	360.4 a	32.67 a	1.16 a	1.31 a

CCF1: continuous crops fertilized with half the recommended P and K rates; CCF2: continuous crops fertilized with the full recommended P and K rates; ICLF1: integrated crop–livestock with half the recommended P and K rates; ICLF2: integrated crop–livestock at full recommended P and K rates. Means followed by the same letter in the columns did not differ by the Tukey test at 5% probability.

**Table 5:** Cumulative N_2_O emissions from soil under soybeans under four production systems in the growing seasons 2015/2016 and 2016/2017.

Cumulative N_2_O Emissions		
Management System	2015/2016	2016/2017
kg ha^−1^		
CCF1	0.71 a	0.16 a
CCF2	0.85 a	0.24 a
ICLF1	0.36 b	0.11 a
ICLF2	0.51 b	0.18 a

CCF1: continuous crops fertilized with half the recommended P and K rates; CCF2: continuous crops fertilized with the full recommended P and K rates; ICLF1: integrated crop–livestock with half the recommended P and K rates; ICLF2: integrated crop–livestock at full recommended P and K rates. Means followed by the same letter in the columns did not differ by the Tukey test at 5% probability.

**Table 6:** Sequence of crop systems over 26 experimental years prior to soil sampling.

Management System ^a^	Annual Sequence of Crops (1991–2017 ^b^)
Continuous crop (CCF1 ^c^)	S-S-M-S-M-S-M-S-S-Pg-S-Pg-S-S-Sb-S-S-M-S-M-S-M-S-S-Sb-M-S-S
Continuous crop (CCF2)	S-S-M-S-M-S-M-S-S-Pg-S-Pg-S-S-Sb-S-S-M-S-M-S-M-S-S-Sb-M-S-S
Crop–livestock (ICLF1)	S-S-M-S-Ag-Ag-Ag-Ag-S-Pg-S-Pg-S/Ub-Ub-Ub-S-Pg-P-P-S-P-S-Pg/P-M-S-S
Crop–livestock (ICLF2)	S-S-M-S-Ag-Ag-Ag-Ag-S-Pg-S-Pg-S/Ub-Ub-Ub-S-Pg-P-P-S-P-S-Pg/P-M-S-S

^a^ CCF1: continuous crops fertilized with half the recommended P and K rates; CCF2: continuous crops fertilized with the full recommended P and K rates; ICLF1: integrated crop–livestock with half the recommended P and K rates; ICLF2: integrated crop–livestock at full recommended P and K rates. ^b^ Ag—*Andropogon gayanus*; M—maize (Zea mays); S—soybean (*Glycine max*); P—*Urochloa brizantha* cv BRS Piatã; Sb—*Sorghum bicolor*; Pg—*Pennisetum glaucum*; Ub—*Urochloa brizantha* cv. Marandu. ^c^ Fertilizers applied in the period: CCF1 (7.15 t ha^−1^ of lime; 500.5 kg N ha^−1^; 1630 kg P_2_O_5_ ha^−1^; 1250 kg K_2_O ha^−1^; 42.7 kg ha^−1^ of micronutrients; and 1.5 t ha^−1^ of gypsum); CCF2 (10.6 t ha^−1^ of lime; 680.5 kg N ha^−1^; 2561 kg P_2_O_5_ ha^−1^; 2146 kg K_2_O ha^−1^; 85 kg ha^−1^ of micronutrients; and 4.3 t ha^−1^ of gypsum); ICLF1 (7.13 t ha^−1^ of lime, 412,5 kg N ha^−1^, 1213 kg ha^−1^ of P_2_O_5_, 903 kg ha^−1^ of K_2_O, 31.5 kg ha^−1^ of micronutrients; and 1.5 t ha^−1^ of gypsum); ICLF2 (10.6 t ha^−1^ of lime; 477.5 kg N ha^−1^; 1793 kg P_2_O_5_ ha^−1^; 1438 kg K_2_O ha^−1^; 63 kg ha^−1^ of micronutrients; and 4.3 t ha^−1^ of gypsum).

**Table 7:** Soil chemical analysis of the experimental area (0–10 cm layer).

Management System	Al	Ca	H + Al	SOM	K	P	pH
	cmol_c_ dm^−3^			%	mg L^−1^		
CCF1	0.044	4.06	3.81	4.06	111.25	3.82	5.65
CCF2	0.020	5.49	3.02	4.59	139	7.81	5.51
ICLF1	0.032	3.56	4.29	3.78	108	12.30	5.35
ICLF2	0.028	5.36	3.12	3.58	169.75	27.62	5.54

CCF1: continuous crops fertilized with half the recommended P and K rates; CCF2: continuous crops fertilized with the full recommended P and K rates; ICLF1: integrated crop–livestock with half the recommended P and K rates; ICLF2: integrated crop–livestock at full recommended P and K rates. SOM: soil organic matter.

## Data Availability

The data presented in this study are available on request from the corresponding author.
